# Pharmacological Modulation of the Mitochondrial Electron Transport Chain in Paclitaxel-Induced Painful Peripheral Neuropathy

**DOI:** 10.1016/j.jpain.2015.06.008

**Published:** 2015-10

**Authors:** Lisa A. Griffiths, Sarah J.L. Flatters

**Affiliations:** Wolfson Centre for Age-Related Diseases, Institute of Psychiatry, Psychology and Neuroscience, King's College London, London, United Kingdom

**Keywords:** Mitochondria, pain, chemotherapy-induced neuropathy, Taxol, paclitaxel

## Abstract

Paclitaxel is an effective first-line chemotherapeutic with the major dose-limiting side effect of painful neuropathy. Mitochondrial dysfunction and oxidative stress have been implicated in paclitaxel-induced painful neuropathy. Here we show the effects of pharmacological modulation of mitochondrial sites that produce reactive oxygen species using systemic rotenone (complex I inhibitor) or antimycin A (complex III inhibitor) on the maintenance and development of paclitaxel-induced mechanical hypersensitivity in adult male Sprague Dawley rats. The maximally tolerated dose (5 mg/kg) of rotenone inhibited established paclitaxel-induced mechanical hypersensitivity. However, some of these inhibitory effects coincided with decreased motor coordination; 3 mg/kg rotenone also significantly attenuated established paclitaxel-induced mechanical hypersensitivity without any motor impairment. The maximally tolerated dose (.6 mg/kg) of antimycin A reversed established paclitaxel-induced mechanical hypersensitivity without any motor impairment. Seven daily doses of systemic rotenone or antimycin A were given either after paclitaxel administration or before and during paclitaxel administration. Rotenone had no significant effect on the development of paclitaxel-induced mechanical hypersensitivity. However, antimycin A significantly inhibited the development of paclitaxel-induced mechanical hypersensitivity when given before and during paclitaxel administration but had no effect when given after paclitaxel administration. These studies provide further evidence of paclitaxel-evoked mitochondrial dysfunction in vivo, suggesting that complex III activity is instrumental in paclitaxel-induced pain.

**Perspective:**

This study provides further in vivo evidence that mitochondrial dysfunction is a key contributor to the development and maintenance of chemotherapy-induced painful neuropathy. This work also indicates that selective modulation of the electron transport chain can induce antinociceptive effects in a preclinical model of paclitaxel-induced pain.

## Highlights

• Complex I inhibition attenuated established paclitaxel-induced pain behavior.
• Complex III inhibition reversed established paclitaxel-induced pain behavior.
• Only complex III inhibition attenuated the development of paclitaxel-induced pain.
• Our data provide new evidence for paclitaxel-evoked mitochondrial dysfunction in vivo.

Paclitaxel, a taxane-derived chemotherapeutic, is a first-line treatment for solid tumors, particularly breast and ovarian carcinomas. Its primary anticancer action occurs via disruption of the mitotic spindle and microtubule dynamics resulting in cycle arrest and apoptosis.^13,17,37^ The major dose-limiting adverse effect of paclitaxel treatment is a peripheral, predominantly sensory, neuropathy that occurs in a stocking-and-glove distribution.^28,38^ Patients typically report numbness, tingling, spontaneous pain, and evoked pain to mechanical and cold stimuli.^3,6,12^ The severity of symptoms depends on various factors, including cumulative dose^33^ and the presence of comorbidities associated with increased risk of neuropathy, such as diabetes.^5^ Unfortunately, patients may continue to experience symptoms for months or years after cessation of paclitaxel.^3,6,45^ Treatment options for chemotherapy-induced painful peripheral neuropathies are very limited. Most analgesics with established efficacy in neuropathic pain are not effective in patients with chemotherapy-induced painful peripheral neuropathy.^19,20,34,35^ However, duloxetine was recently reported to significantly reduce pain scores compared with placebo in this patient population.^41^ Differential sensitivity of chemotherapy-induced pain to otherwise effective analgesics could potentially relate to differences in the causal mechanisms of neuropathic pain states.

Paclitaxel-induced painful peripheral neuropathy has been accurately modeled in rats using low doses of systemic paclitaxel on alternate days to mimic cycles of chemotherapy, which evokes hypersensitivity to mechanical and cold stimuli.^8,9^ In this translational rat model of paclitaxel-induced painful peripheral neuropathy, increased incidence of atypical (swollen and vacuolated) mitochondria was observed in peripheral sensory nerves in the absence of mid-axonal degeneration.^10^ Furthermore, these atypical changes in neuronal mitochondria correlate with the presence of paclitaxel-induced mechanical hypersensitivity,^10^ suggesting a causal role of mitochondrial dysfunction in paclitaxel-induced painful peripheral neuropathy. The presence of swollen and vacuolated mitochondria indicates mitochondrial dysfunction but does not indicate the nature of the mitochondrial dysfunction given the multiple functions that mitochondria perform. Mitochondria are a major cellular source of reactive oxygen species (ROS).^29^ ROS are free radicals and reactive molecules that are derived from molecular oxygen; examples include superoxide, hydroxyl radical, nitric oxide, and hydrogen peroxide. ROS can react with many different substrates and with each other; for example superoxide and nitric oxide react to form peroxynitrite.^30^ Several studies have provided evidence that ROS are involved in nerve injury–induced and inflammatory pain.^16,21,22,26,31,39,40^ Moreover, systemic administration of a nonspecific ROS scavenger prevented the development of paclitaxel-induced mechanical hypersensitivity and reversed established paclitaxel-induced mechanical and cold hypersensitivities.^8,23^ In addition, peroxynitrite decomposition catalysts were reported to prevent and reverse paclitaxel-induced mechanical hypersensitivity.^7^

Embedded within the inner mitochondrial membrane are the protein complexes of the electron transport chain, which are key components of oxidative phosphorylation. Complex I^44^ and complex III^43^ are known sources of ROS as a consequence of electron transfer. In pathological states, the amount of ROS produced at these complexes may increase beyond the antioxidant capacity of the cell, resulting in oxidative stress. In our previous study,^8^ we established the importance of ROS in vivo to the development and maintenance of paclitaxel-induced pain. Here, we have used selective pharmacological inhibitors of complex I (rotenone) and complex III (antimycin A) to assess the effects of modulation of the electron transport chain on the development and maintenance of paclitaxel-induced painful peripheral neuropathy. This work was previously presented in abstract form.^15^

## Methods

### Animals

Adult male Sprague Dawley rats (starting weight 170–220 g; Harlan, Oxford, UK) were housed in groups of 4 in plastic cages with sawdust bedding and environmental enrichment materials, in a room only containing rats. Bedding/cages were changed twice a week. Artificial light was provided on a 12-hour light-dark cycle (lights on at 7 am), and standard rat chow and water were available ad libitum. Rats were habituated to the testing environment for approximately 30 minutes on 3 separate occasions before baseline mechanical sensitivity testing. Throughout all studies, rats were routinely checked visually and weighed to ensure good health. Health status before treatment was normal. All studies were carried out in accordance with the UK Animals (Scientific Procedures) Act (1986) and the ethical guidelines issued by the International Association for the Study of Pain.^48^

### Assessment of Mechanical Hypersensitivity

Rats were placed in an elevated apparatus consisting of a wire-rung floor (spaced 8 mm apart), with each rat separated by acrylic dividers (box dimensions 15  cm × 16  cm × 21 cm). Rats were allowed to acclimatize for 5 to 10 minutes. Testing commenced when the animal was alert, settled, and weight-bearing equally on all 4 paws. Von Frey filaments (Touch-Test Sensory Evaluators, Linton Instrumentation, Norfolk, UK) of 4 g, 8 g, and 15 g bending forces were used in ascending order of force. The filament was applied 5 times, in a pseudorandom order, to the midplantar surface of each hind paw, and each stimulus was held for 5 seconds. The number of withdrawal responses for the 2 hind paws were added together to give a score out of a maximum of 10 withdrawals for each of the 3 von Frey filaments. After habituation, 3 baseline measurements of mechanical sensitivity were taken before commencing paclitaxel administration. Mechanical sensitivity was assessed in the morning (8–11 am).

### Assessment of Motor Coordination

Motor coordination was assessed using an accelerating Rota-rod (Ugo-Basile, Monvalle, Varese, Italy) and catalepsy ring apparatus. The initial speed of the Rota-rod was set at 5 rotations per minute (rpm) and gradually increased from 5 to 40 rpm over 5 minutes (300 seconds). The latency (in seconds) of the rats falling off the Rota-rod onto the sensor platform was recorded. Two or 3 training sessions were given (until all rats were able to remain on the apparatus for 180 seconds or more), followed by 1 baseline measurement before commencing paclitaxel administration. All rats were tested at a similar time on each testing day (late morning, after testing for mechanical hypersensitivity). After testing on the accelerating Rota-rod, catalepsy was assessed using a ring apparatus (Fisherbrand, Loughborough, UK) as previously described.^32^ Each rat was placed onto a horizontal steel ring, 12.5 cm in diameter, and elevated approximately 9 cm above a platform. The rat was positioned so that all 4 paws were gripping the ring. A timer was stopped when the rat put at least 1 paw on the platform. The time spent immobile on the ring, expressed as latency (seconds), was noted. Two training sessions were given, followed by one baseline measurement before commencing paclitaxel administration.

### Drug Administration

#### Paclitaxel

After habituation and baseline mechanical sensitivity testing, rats received intraperitoneal (i.p.) injections of 2 mg/kg paclitaxel on 4 alternate days (days 0, 2, 4, and 6) as previously described.^8-11^ Paclitaxel concentration for solution for infusion (6 mg/mL; CP Pharmaceuticals Ltd/Actavis, Devon, UK) was diluted with .9% sterile saline (Fresenius Kabi, Manor Park, UK) and injected at 2 mg/mL/kg body weight. Injections were performed in the morning, and rats were immediately returned to their home cages afterward.

#### Inhibitors of Complex I (Rotenone) and Complex III (Antimycin A)

Rotenone and antimycin A (Sigma-Aldrich, Dorset, UK) were dissolved in Miglyol 812N (Azelis, Hertford, UK) and sonicated for 15 to 60 minutes resulting in clear colorless solutions. Rotenone or antimycin A was administered i.p. (for systemic exposure) at 1 mL/kg body weight in either treatment (see section on Effects of Complex I/III Inhibition on Established Paclitaxel-Induced Mechanical Hypersensitivity) or prophylactic dosing paradigms (see section on Effects of Complex I/III Inhibition on the Development of Paclitaxel-Induced Mechanical Hypersensitivity) to test if the inhibition of complex I or III could inhibit established paclitaxel-induced pain or prevent the development of paclitaxel-induced pain, respectively. Unless otherwise stated, injections of complex inhibitors were performed in the morning, and rats were immediately returned to their home cages afterward. In initial studies, 1 mg/kg and 2 mg/kg rotenone were used. The choice of these doses was based on reports of repeated dosing with 3 mg/kg rotenone to model Parkinson disease.^4^ In our pilot studies of drug tolerability, we found 5 mg/kg rotenone was the maximally tolerated dose when given as a single bolus i.p. injection in naive rats. The median lethal dose of antimycin A in adult rats was previously shown to be .81 mg/kg.^36^ In our pilot studies, we found .6 mg/kg antimycin A was the maximally tolerated dose when given as a single bolus i.p. injection in naive rats. Because of toxicity concerns with repeated daily dosing, 2 mg/kg rotenone and .4 mg/kg antimycin A were the maximum doses used in prophylactic studies.

### Blinding and Randomization

In all studies, rats were split into groups of equal mechanical sensitivity based on their average baseline or post-paclitaxel responses. Drug treatments were randomized within the groups of rats being tested in a given session. Therefore, a concurrent vehicle-treated group was present throughout all experiments to minimize extraneous variables on behavioral responses such as time of day. All behavioral testing was carried out by a single experimenter (L.A.G.), who was blinded to the treatment group. For studies on established mechanical hypersensitivity, drug/vehicle administration was performed by another scientist (S.J.L.F.). For prophylactic studies, the experimenter (L.A.G.) carried out all injections but the drug vials were relabeled by a third party before drug administration. In the prophylactic studies, there was a period of overlap between drug administration and behavioral testing. During this period, the cages were relabeled by a third party before behavioral testing, which further disguised the identity of the treatment received by each rat during testing. When drug administration was complete, the rats were renumbered by a third party for the remaining duration of the experiment. At the end of the studies, the identity of the treatment each rat received was revealed for data analysis. Sample size was determined based on extensive previous studies using rats with paclitaxel-induced painful neuropathy.^8-11^

### Effects of Complex I/III Inhibition on Established Paclitaxel-Induced Mechanical Hypersensitivity

The effects of single bolus doses of rotenone or antimycin A on established paclitaxel-induced mechanical hypersensitivity were assessed in separate experiments. On day 26/28 after paclitaxel initiation, after assessment of mechanical sensitivity, rats received an i.p. injection of either rotenone (1 mg/kg or 2 mg/kg) or vehicle (n = 8 per group). Neither 1 mg/kg nor 2 mg/kg rotenone had any effect on mechanical hypersensitivity at 1, 3, or 24 hours after injection (data not shown). In another experiment, on day 28/29 after paclitaxel initiation, after assessment of mechanical sensitivity, rats received an i.p. injection of either rotenone (3 mg/kg or 5 mg/kg, n = 7) or vehicle (n = 6). The effects of these higher doses of rotenone were assessed at 1, 3, and 24 hours after injection. At the 24-hour time point, 2 rats were obviously sedated and were excluded from testing. At the end of the experiment, these 2 rats were found to have received 5 mg/kg rotenone. On day 33 after paclitaxel initiation, after assessment of mechanical sensitivity, rats received an i.p. injection of either antimycin A (.2 mg/kg or .4 mg/kg) or vehicle (n = 8 per group). Neither .2 mg/kg nor .4 mg/kg antimycin A had any effect on mechanical hypersensitivity at 1, 3, and 24 hours after injection (data not shown). In a further experiment, also on day 33 after paclitaxel initiation, after assessment of mechanical sensitivity, rats received an i.p. injection of either .6 mg/kg antimycin A or vehicle (n = 8 per group). The effects of this higher dose of antimycin A were assessed at 1, 3, and 24 hours after injection.

### Effects of Complex I/III Inhibition on the Development of Paclitaxel-Induced Mechanical Hypersensitivity

The effects of repeated dosing of rotenone or antimycin A on the development of paclitaxel-induced mechanical hypersensitivity were assessed in separate experiments using 2 different prophylactic dosing paradigms. Complex inhibitors or vehicle were administered once daily for 7 days to rats either after paclitaxel treatment on days 7 to 13, or before and during paclitaxel treatment on days −1 to 5 (paclitaxel was given on days 0, 2, 4, and 6, as described earlier).

#### Prophylactic Complex I/III Inhibition (Days 7 to 13)

After the final baseline test for mechanical sensitivity (day 0), all rats received paclitaxel as described earlier. On day 7, after assessment of mechanical sensitivity, rats were allocated into 3 groups of equal sensitivity (n = 8 per group). In an experiment, rats then received either rotenone (1 mg/kg or 2 mg/kg i.p.) or vehicle once daily on days 7 to 13. In a similar separate experiment, on day 7, rats then received either antimycin A (.2 mg/kg or .4 mg/kg i.p.) or vehicle once daily on days 7 to 13. During this experiment, 1 rat died part way through the drug administration protocol. In addition, an error in blinding and randomization procedures occurred, so 8 rats that had been incorrectly assigned were removed from the experiment. The antimycin A (.2 mg/kg or .4 mg/kg i.p.)/vehicle experiment was repeated at a later date (n = 4 per group) and all 12 animals survived. Thus, we pooled the data from both blinded randomized experiments resulting in n = 9 per group. In both prophylactic studies with complex inhibitor/vehicle administration on days 7 to 13, rats were assessed for the development of mechanical hypersensitivity in the morning on days 10, 12, 14, 18, 23, 28, 33, 38, and 44.

#### Prophylactic Complex I/III Inhibition (Days −1 to 5)

Only the highest doses of rotenone and antimycin A used in the prophylactic dosing paradigm (days 7–13) were used for these experiments to minimize animal usage in accordance with the 3 Rs principles of animal research (Replacement, Reduction, and Refinement). After the final baseline assessment of mechanical sensitivity (day −1), rats were split into 2 groups of equal sensitivity (n = 8 per group). In 1 experiment, rats received either i.p. 2 mg/kg rotenone or vehicle. In a similar separate experiment, on day −1, rats then received either i.p. .4 mg/kg antimycin A or vehicle. In both experiments, the following morning (day 0) after the first injection of complex inhibitor or vehicle, rats were assessed for the acute effects of complex I/III inhibition on mechanical sensitivity. Rats were then injected with 2 mg/kg paclitaxel, and 5 to 6 hours later the second injection of complex I/III inhibitor was given. Rats were injected once daily with complex I/III inhibitor or vehicle on days −1 to 5 and paclitaxel on days 0, 2, 4, and 6. When complex inhibitor/vehicle and paclitaxel were administered on the same day, paclitaxel was injected between 11 am and 12 pm, followed by complex inhibitor/vehicle administration between 5 pm and 6 pm. On the morning of day 6, after the last injection of complex inhibitor/vehicle and before paclitaxel administration, rats were assessed for mechanical hypersensitivity to examine any effects of cumulative dosing. In the initial antimycin A/vehicle experiment, 5 rats died after the last injection on day 5. After unblinding at the end of the experiment, all 5 rats were found to have received antimycin A. The reason for these deaths is unclear, although it may have been due to a lower starting weight (208–224 g) and, compared with vehicle-treated rats, minimal weight gain during the period of antimycin A administration. The .4 mg/kg antimycin A and vehicle experiment was repeated at a later date (n = 6 per group) with slightly heavier rats (240–255 g), and all 12 animals survived. Thus, we pooled the data from both blinded randomized experiments resulting in n = 9 for antimycin A and n = 14 for vehicle. In both prophylactic studies with complex inhibitor/vehicle administration on days −1 to 5, rats were also assessed for the development of mechanical hypersensitivity in the morning on days 10, 14, 18, 23, 28, 33, 38, and 44.

### Effects of Complex I/III Inhibition on Motor Coordination

Rotenone is used experimentally to generate motor deficits modeling Parkinson disease^4^ in a dosing regimen of up to 3 mg/kg/day for up to 60 days, with motor deficits appearing after 6 days. Therefore, we tested the effects of complex inhibitors on motor coordination to assess if observed drug effects on paclitaxel-induced mechanical hypersensitivity were due to sensory changes alone. Naive rats, weight-matched to those in established pain studies (300–330 g), received i.p. 5 mg/kg rotenone, .6 mg/kg antimycin A, or vehicle (n = 6 per group), and in a separate study either i.p. 3 mg/kg rotenone or vehicle (n = 6 per group). Motor coordination using accelerating Rota-rod and catalepsy ring (see section on Assessment of Motor Coordination) was then assessed at 1, 3, and 24 hours after injection of complex inhibitor/vehicle. There was no difference in responses between groups before receiving the injection of complex inhibitor/vehicle, so these data were pooled for baseline responses. The effects of repeated daily dosing for 7 days of rotenone and antimycin A on motor coordination were also assessed. During the prophylactic complex I/III inhibition experiments (see section on Effects of Complex I/III Inhibition on the Development of Paclitaxel-Induced Mechanical Hypersensitivity), performance on the Rota-rod and catalepsy ring was assessed on days −1 (baseline), 0, 6, 14, 23, 33, and 44 after testing for mechanical hypersensitivity. As previously described, paclitaxel-treated rats received i.p. 2 mg/kg rotenone or vehicle (n = 8 per group), and in a separate experiment either i.p. .4 mg/kg antimycin A or vehicle (n = 9–14 per group). For ease of illustration, we pooled the vehicle group data from both blinded randomized experiments, resulting in n = 8 for rotenone, n = 9 for antimycin A, and n = 22 for vehicle. For statistical comparisons between the effects of rotenone or antimycin A administration and vehicle treatment, data from the concurrent vehicle-treated group in each experiment were used.

### Statistics

All analyses were carried out using GraphPad Prism 6 or GraphPad InStat 3 for Windows (GraphPad Software, Inc, La Jolla, CA). To assess for the presence of paclitaxel-induced mechanical hypersensitivity in established pain studies, a paired 1-tailed t-test was carried out. For established pain studies with 3 treatment groups, a 1-way analysis of variance (ANOVA) with Dunnett post hoc comparison with vehicle control at each time point was carried out. For established pain studies with 2 treatment groups, unpaired 2-tailed t-tests with Bonferroni correction were used to compare the effects of complex inhibition with vehicle treatment. Paired 2-tailed t-tests were used to compare responses after complex inhibition to pre-paclitaxel baseline responses to assess if established paclitaxel-induced mechanical hypersensitivity had been reversed and to test if complex inhibition significantly attenuated responses beyond pre-paclitaxel response levels. One-way repeated measures (RM) ANOVA with Dunnett post hoc comparison with pre-paclitaxel baseline responses was used to test for significant development of paclitaxel-induced mechanical hypersensitivity. To assess the acute effects of complex inhibition on mechanical hypersensitivity (prophylactic paradigm days −1 to 5), paired 1-tailed t-tests were performed comparing day −1 with baseline responses. The effect of complex inhibition on the development of paclitaxel-induced mechanical hypersensitivity was also assessed using an area under the curve analysis on data collected from day 10 onward, with unpaired 2-tailed t-tests to compare vehicle with drug treatment group. To assess the acute effects of complex inhibition on motor coordination of naive rats, a 1-way ANOVA with Dunnett post hoc comparison with vehicle control at each time point was performed when there were 3 treatment groups, and unpaired 2-tailed t-tests with Bonferroni correction were used to compare the effects of complex inhibition with vehicle treatment in experiments with 2 groups (data not shown). To assess the effects of multiple injections of complex inhibitors on motor coordination in paclitaxel-treated rats, 2-way repeated measures ANOVA with Bonferroni post hoc comparison with vehicle control at each time point was performed. All animals were used in the statistical analyses unless otherwise stated. Data are presented as mean ± standard error of the mean (SEM). Statistical significance was accepted at *P* < .05. No further distinction has been made when *P* < .01 or *P* < .001 and is denoted on figures as *P* < .05.

## Results

### Effect of Complex I Inhibition on Established Paclitaxel-Induced Mechanical Hypersensitivity

**Figure 1:**
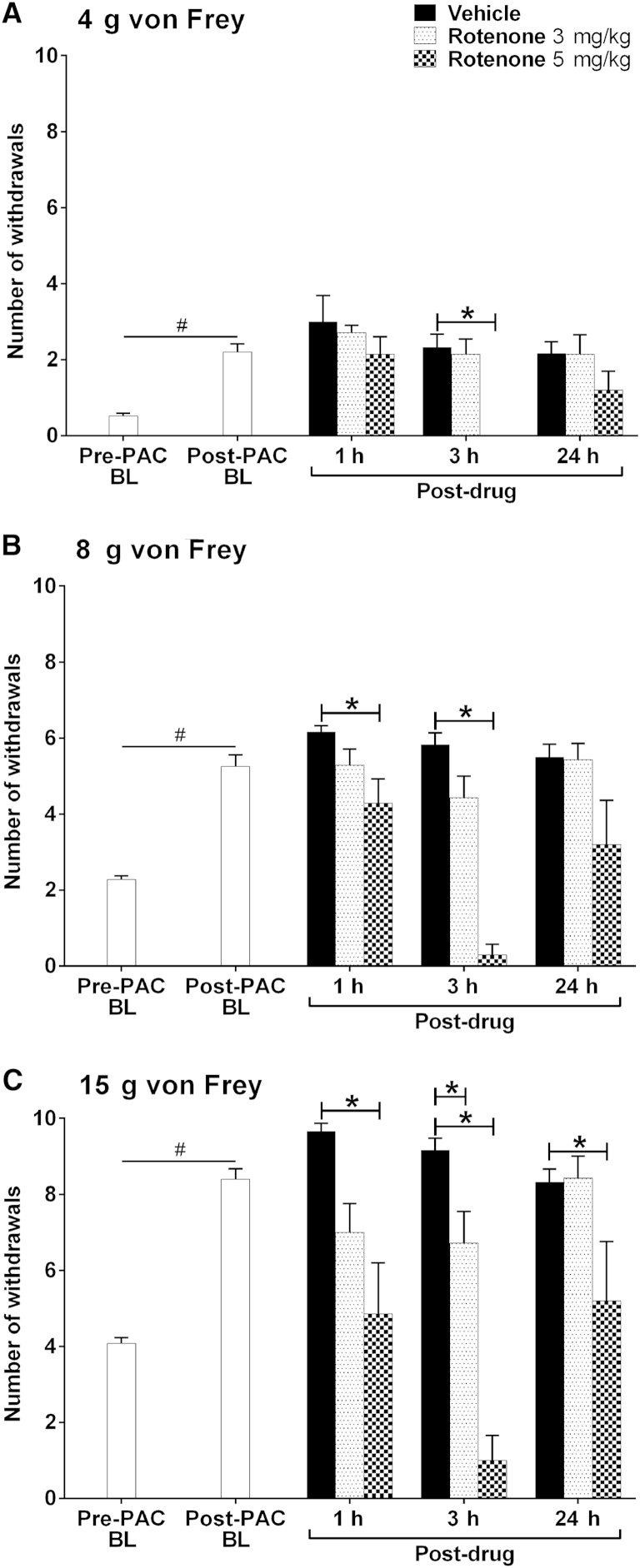
Effect of complex I inhibition on established paclitaxel-induced mechanical hypersensitivity. **(A–C)** The effect of rotenone on withdrawal responses to 4 g, 8 g, and 15 g von Frey, respectively. Graphs show the mean ± SEM of the number of withdrawals before paclitaxel (Pre-PAC BL, n = 20), after paclitaxel before rotenone/vehicle (Post-PAC BL, n = 20), then 1, 3, and 24 hours after injection of rotenone (3 mg/kg or 5 mg/kg) or vehicle (Post-drug, n = 6–7 per group, except n = 5 at 24 hours for 5 mg/kg rotenone). #*P* < .05, 1-tailed paired t-tests comparing with pre-paclitaxel baseline responses. *P < .05, 1-way ANOVA with Dunnett post hoc comparing with a concurrent vehicle-treated group. In a separate experiment, 1 mg/kg and 2 mg/kg rotenone had no effect on paclitaxel-induced mechanical hypersensitivity (data not shown).

Four systemic injections of 2 mg/kg paclitaxel resulted in a robust mechanical hypersensitivity on day 28/29 after paclitaxel initiation (Fig 1, *P* < .05, 1-tailed paired t-tests) as previously described.^8-10^ Administration of a single dose of either 1 or 2 mg/kg rotenone had no effect on paclitaxel-induced mechanical hypersensitivity at 1, 3, or 24 hours after administration (data not shown). Three mg/kg rotenone significantly attenuated responses to von Frey 15 g at 3 hours after administration compared with the concurrent vehicle-treated group (Fig 1C, *P* < .05, 1-way ANOVA with Dunnett post hoc analysis). Some inhibitory effects of 3 mg/kg rotenone were also observed at 1 and 3 hours after administration with von Frey 15 g and von Frey 8 g responses, but these were not statistically significant (Figs 1B and 1C). One hour after 5 mg/kg rotenone administration, responses to von Frey 8 g and 15 g were significantly inhibited compared with the concurrent vehicle-treated group (Figs 1B and 1C, *P* < .05 1-way ANOVA with Dunnett post hoc analysis). Three hours after 5 mg/kg rotenone administration, responses to all von Freys were profoundly inhibited compared with the concurrent vehicle-treated group (Fig 1, *P* < .05, 1-way ANOVA with Dunnett post hoc analysis). The inhibition by 5 mg/kg rotenone at this 3-hour time point was a complete reversal of paclitaxel-induced mechanical hypersensitivity but also a significant inhibition beyond pre-paclitaxel baseline responses (*P* < .05, 2-tailed paired t-tests). Twenty-four hours after rotenone administration, the observed inhibitory effects of rotenone had largely disappeared. There was no difference between rotenone-treated groups and the vehicle group, with the exception of 5 mg/kg rotenone on von Frey 15 g responses.

### Effect of Complex III Inhibition on Established Mechanical Hypersensitivity

**Figure 2:**
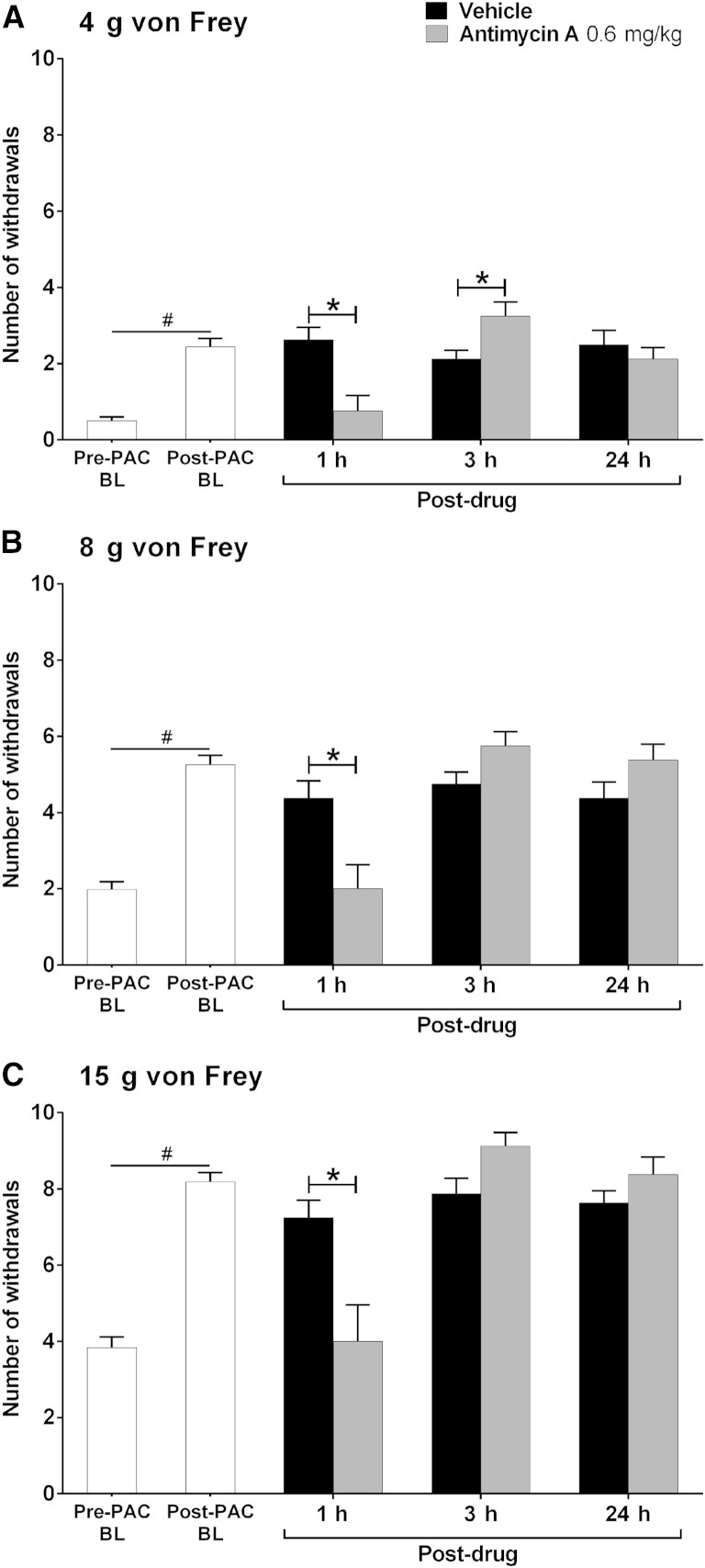
Effect of complex III inhibition on established paclitaxel-induced mechanical hypersensitivity. **(A–C)** The effect of antimycin A on withdrawal responses to 4 g, 8 g, and 15 g von Frey, respectively. Graphs show the mean ± SEM of the number of withdrawals before paclitaxel (Pre-PAC BL, n = 16), after paclitaxel before rotenone/vehicle (Post-PAC BL, n = 16), then 1, 3, and 24 hours after injection of .6 mg/kg antimycin A or vehicle (Post-drug, n = 8 per group). #*P* < .05, 1-tailed paired t-tests comparing with pre-paclitaxel baseline responses. *P < .05, 2-tailed unpaired t-tests with Bonferroni correction comparing with a concurrent vehicle-treated group. In a separate experiment, .2 mg/kg and .4 mg/kg antimycin A had no effect on paclitaxel-induced mechanical hypersensitivity (data not shown).

We have consistently found with multiple rat cohorts that 4 systemic injections of 2 mg/kg paclitaxel on alternate days (0, 2, 4, and 6) evokes a marked long-lasting mechanical hypersensitivity, which reaches its maximum around day 27, remains high for several weeks, and finally resolves approximately 6 months after paclitaxel initiation (data not shown). Thus, as expected, a marked paclitaxel-induced mechanical hypersensitivity was observed at day 33 after paclitaxel initiation (Fig 2, *P* < .05, 1-tailed paired t-tests) that was of similar magnitude to that observed at day 28/29 (Fig 1).

Administration of a single dose of either .2 or .4 mg/kg antimycin A had no effect on paclitaxel-induced mechanical hypersensitivity at 1, 3, or 24 hours after administration (data not shown). One hour after .6 mg/kg antimycin A administration, responses to all von Frey hairs were significantly inhibited compared with the concurrent vehicle-treated group (Fig 2, *P* < .05, 2-tailed unpaired t-tests with Bonferroni correction). This observed inhibition evoked by .6 mg/kg antimycin A constituted a complete reversal of paclitaxel-induced mechanical hypersensitivity as there was no significant difference between post-antimycin A responses and pre-paclitaxel responses to all von Freys (Fig 2, 2-tailed paired t-tests). Three hours after .6 mg/kg antimycin A, there was a small but significant increase in responses to von Frey 4 g compared to vehicle (Fig 2A, *P* < .05, 2-tailed unpaired t-test with Bonferroni correction) but no effect on responses to von Frey 8 g and 15 g (Figs 2B and 2C). No persistent effect of antimycin A was observed at 24 hours after administration, with withdrawal responses to all von Freys returning to post-paclitaxel/pre-antimycin A response levels.

### Effect of Complex I Inhibition on Development of Paclitaxel-Induced Mechanical Hypersensitivity

**Figure 3:**
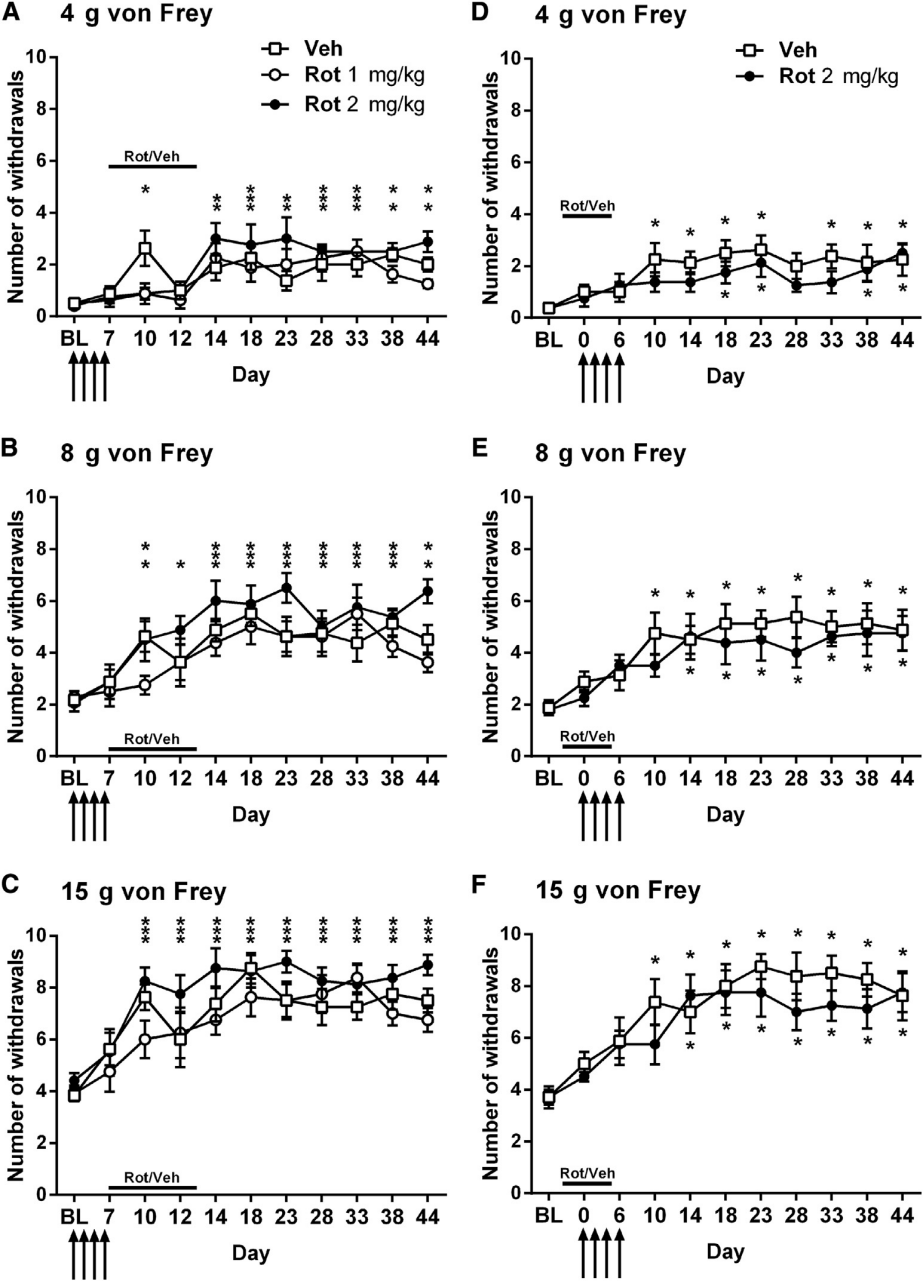
Effect of complex I inhibition on the development of paclitaxel-induced mechanical hypersensitivity. All graphs show the mean ± SEM of the number of withdrawals to von Frey stimulation. **(A–C)** The effect of daily dosing of rotenone (1 mg/kg or 2 mg/kg) on days 7 to 13 (after paclitaxel therapy), on withdrawal responses to 4 g, 8 g, and 15 g von Frey, respectively, up to day 44 after paclitaxel initiation. **(D–F)** The effect of daily dosing of rotenone (2 mg/kg) on days −1 to 5 (before and during paclitaxel therapy), on withdrawal responses to 4 g, 8 g, and 15 g von Frey, respectively, up to day 44 after paclitaxel initiation. Solid line indicates the period of rotenone/vehicle administration. Arrows indicate 4 injections of 2 mg/kg paclitaxel on days 0, 2, 4, and 6. In all cases, *P < .05, 1-way RM ANOVA with Dunnett post hoc comparing to pre-paclitaxel baseline responses indicating the occurrence of significant paclitaxel-induced mechanical hypersensitivity; n = 8 per group. **(A–C)** Asterisks are in the following order: Veh (upper row), 1 mg/kg Rot (center), 2 mg/kg Rot (lower). ** or *** indicates that 2 or 3 groups display significant paclitaxel-induced mechanical hypersensitivity at that time point. Veh, vehicle; Rot, rotenone.

We have previously found that scavenging ROS in vivo not only markedly inhibited established paclitaxel-induced mechanical hypersensitivity but could also prevent the development of paclitaxel-induced mechanical hypersensitivity.^8^ Given these previous findings and the observed inhibitory effects of complex I inhibition on paclitaxel-induced mechanical hypersensitivity, we examined the effects of complex I inhibition on the development of paclitaxel-induced mechanical hypersensitivity. Because of concerns regarding potential motor effects of prolonged daily dosing with rotenone, we limited daily dosing to 7 consecutive days. In separate experiments, rotenone was administered either on days 7 to 13 or on days −1 to 5 to examine the effects of complex I inhibition on the development of paclitaxel-induced mechanical hypersensitivity after paclitaxel therapy or before/during paclitaxel therapy, respectively (Fig 3).

Administration of 7 daily doses of 1 or 2 mg/kg rotenone on days 7 to 13, commencing the day after the final injection of paclitaxel, had no significant effect on the eventual development of paclitaxel-induced mechanical hypersensitivity (Figs 3A–3C). Overall, from day 14 onward, significant paclitaxel-induced mechanical hypersensitivity compared with pre-paclitaxel baseline responses to all von Freys was observed in both the rotenone- and vehicle-treated groups (Figs 3A–3C, *P* < .05, 1-way RM ANOVA with Dunnett post hoc analysis). The exceptions to this were von Frey 4 g responses for the vehicle-treated group at days 14 and 23, von Frey 4 g responses for the 1 mg/kg rotenone group at days 38 and 44, and von Frey 8 g responses for the 1 mg/kg rotenone group on day 44. During the period of rotenone/vehicle administration on days 10 and 12, responses to von Frey 4 g were not different from pre-paclitaxel baseline levels for both the 1 mg/kg and 2 mg/kg rotenone groups, whereas significant allodynia was observed in the vehicle group at day 10 (Fig 3A, *P* < .05, 1-way RM ANOVA with Dunnett post hoc analysis). Similarly, responses to von Frey 8 g were also unaltered compared with pre-paclitaxel baseline levels in the 1 mg/kg rotenone group on days 10 and 12, whereas significant hypersensitivity was found in the vehicle group at day 10 (Fig 3A, *P* < .05, 1-way RM ANOVA with Dunnett post hoc analysis). These effects could indicate a slight delay in the full development of paclitaxel-induced mechanical hypersensitivity after rotenone treatment on days 7 to 13.

In a separate experiment, we investigated the effects of rotenone administration before and during paclitaxel therapy with 7 daily doses of 2 mg/kg rotenone on days −1 to 5 (Figs 3D–3F). This rotenone dosing paradigm also had no significant effect on the eventual development of paclitaxel-induced mechanical hypersensitivity. From day 14 onward, both the rotenone- and vehicle-treated groups displayed significant mechanical hypersensitivity compared with pre-paclitaxel baseline responses to all von Freys (Figs 3D–3F, *P* < .05, 1-way RM ANOVA with Dunnett post hoc analysis). Area under the curve analysis from day 10 onward versus pre-paclitaxel baseline values confirmed no overall significant difference in the development of paclitaxel-induced mechanical hypersensitivity between vehicle- and rotenone-treated rats (data not shown). However, also akin to the effects of the day 7 to 13 rotenone dosing paradigm, there was a slight delay in the emergence of mechanical hypersensitivity in the rotenone-treated group. Significant paclitaxel-induced mechanical hypersensitivity was not observed until day 14 in the rotenone-treated group compared with day 10 in the vehicle-treated group (Figs 3D–3F). The experimental design used here enabled the acute nociceptive effects of rotenone to be assessed because the first injection of rotenone was administered on day −1 and the rats were tested on day 0 before the first paclitaxel administration. Rotenone 2 mg/kg did not significantly alter the normal responses to von Frey stimulation (Figs 3D–3F, day 0).

In summary, rotenone administration caused a slight delay in the appearance of paclitaxel-induced mechanical hypersensitivity but did not significantly affect the overall development of paclitaxel-induced mechanical hypersensitivity and its magnitude.

### Effect of Complex III Inhibition on Development of Paclitaxel-Induced Mechanical Hypersensitivity

**Figure 4:**
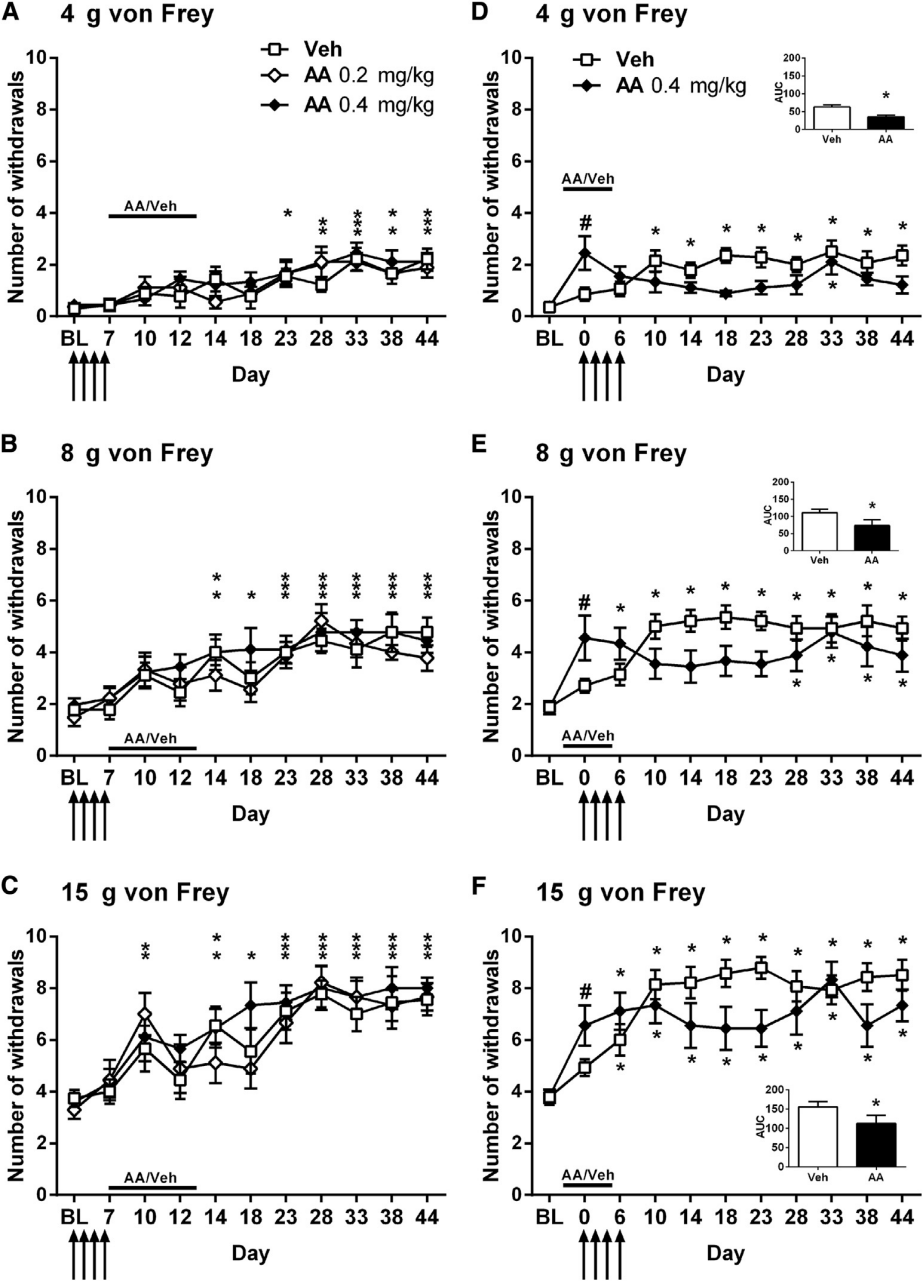
Effect of complex III inhibition on the development of paclitaxel-induced mechanical hypersensitivity. All graphs show the mean ± SEM of the number of withdrawals to von Frey stimulation. **(A–C)** The effect of daily dosing of antimycin A (.2 mg/kg or .4 mg/kg) on days 7 to 13 (after paclitaxel therapy) on withdrawal responses to 4 g, 8 g, and 15 g von Frey, respectively, up to day 44 after paclitaxel initiation. **(D–F)** The effect of daily dosing of .4 mg/kg antimycin A on days −1 to 5 (before and during paclitaxel therapy), on withdrawal responses to 4 g, 8 g, and 15 g von Frey, respectively, up to day 44 after paclitaxel initiation. Solid line indicates periods of antimycin A/vehicle administration. Arrows indicate 4 injections of 2 mg/kg paclitaxel on days 0, 2, 4, and 6. #*P* < .05, 1-tailed paired t-tests compared with baseline responses demonstrating acute pronociceptive effects of antimycin A on normal mechanical responses. In all cases, *P < .05, 1-way repeated measures ANOVA with Dunnett post hoc comparing with pre-paclitaxel baseline responses indicating the occurrence of significant paclitaxel-induced mechanical hypersensitivity; n = 9 to 14 per group. **(A–C)** Asterisks are in the following order: Veh (upper row), .2 mg/kg AA (center), .4 mg/kg AA (lower). ** or *** indicates that 2 or 3 groups display significant paclitaxel-induced mechanical hypersensitivity at that time point. Inserts **(D–F)** show the area under curve analysis of responses at baseline and on days 10 to 44. *P < .05 1-tailed unpaired t-tests. n = 9–14 per group. Veh, vehicle; AA, antimycin A.

To compare the effects of complex III inhibition with complex I inhibition on the development of paclitaxel-induced mechanical hypersensitivity, we performed experiments using antimycin A administered in the same dosing paradigms used in the rotenone experiments described previously. Administration of 7 daily doses of .2 or .4 mg/kg antimycin A on days 7 to 13, commencing the day after the final injection of paclitaxel, had no effect on the eventual development of paclitaxel-induced mechanical hypersensitivity (Figs 4A–4C, *P* < .05, 1-way RM ANOVA with Dunnett post hoc analysis). Overall, from day 23 onward, significant paclitaxel-induced mechanical hypersensitivity compared with pre-paclitaxel baseline responses to all von Freys was observed in both the antimycin A- and vehicle-treated groups (Figs 4A–4C, *P* < .05, 1-way RM ANOVA with Dunnett post hoc analysis). The exceptions to this were von Frey 4 g responses for the vehicle-treated group at day 28, the .2 mg/kg antimycin A group at days 23 and 38, and the .4 mg/kg antimycin A group at day 23. There were significant increases in the number of withdrawal responses to von Frey 8 g and 15 g compared with the baseline responses before day 23 in the time course of this experiment. Rats treated with .4 mg/kg antimycin A displayed significant hypersensitivity to von Frey 8 g and 15 g at days 14 and 18, whereas such effects were seen with the vehicle-treated group on day 14 only. In addition, both antimycin A-treated groups show significantly increased responses to von Frey 15 g at day 10 compared with baseline responses (Fig 4C, *P* < .05, 1-way RM ANOVA with Dunnett post hoc analysis). However, these effects were the only significant effects observed during the antimycin A/vehicle dosing period from days 7 to 13.

In a separate experiment, we investigated the effects of antimycin A administration before and during paclitaxel therapy with 7 daily doses of .4 mg/kg antimycin A on days −1 to 5 (Figs 4D–4F). In contrast to the previous prophylactic dosing (days 7–13) experiment, .4 mg/kg antimycin A significantly delayed and attenuated the development of paclitaxel-induced mechanical hypersensitivity to all von Freys (Figs 4D–4F). The expected significant increase in withdrawal responses to all von Freys compared with the pre-paclitaxel baseline levels was observed in the vehicle-treated group from day 10 onward (Figs 4D–4F, *P* < .05, 1-way RM ANOVA with Dunnett post hoc analysis). In comparison, von Frey 4 g responses were only significantly increased on day 33, and von Frey 8 g responses were significantly increased from day 28 onward in the antimycin A-treated group (Figs 4D and 4E, *P* < .05, 1-way RM ANOVA with Dunnett post hoc analysis). Responses to von Frey 15 g in the antimycin A group were less than those observed in the vehicle group but were significantly increased compared with their pre-paclitaxel baseline (Fig 4F, *P* < .05, 1-way RM ANOVA with Dunnett post hoc analysis). Area under the curve analysis from day 10 onward versus pre-paclitaxel baseline values confirmed a significant overall difference between antimycin A- and vehicle-treated rats in the development of paclitaxel-induced mechanical hypersensitivity to all von Freys (Figs 4D–4F inserts, *P* < .05, 1-tailed paired t-tests). The experimental design used here enabled the acute nociceptive effects of antimycin A to be assessed because the first injection of antimycin A was administered on day −1 and the rats were tested on day 0 before the first paclitaxel administration. A single injection of .4 mg/kg antimycin A resulted in a significant mechanical hypersensitivity to all von Freys compared with baseline response levels (Figs 4D–4F, *P* < .05, 1-tailed paired t-tests).

In summary, antimycin A administration before and during paclitaxel therapy significantly attenuated the overall development of paclitaxel-induced mechanical hypersensitivity. However, antimycin A administration after paclitaxel therapy did not significantly alter the development of paclitaxel-induced mechanical hypersensitivity. In addition, antimycin A significantly increased normal baseline responses to von Frey stimulation, suggesting a differential effect of antimycin A administration in naive versus paclitaxel-treated rats.

### Effect of Complex Inhibitors on Motor Function

**Figure 5:**
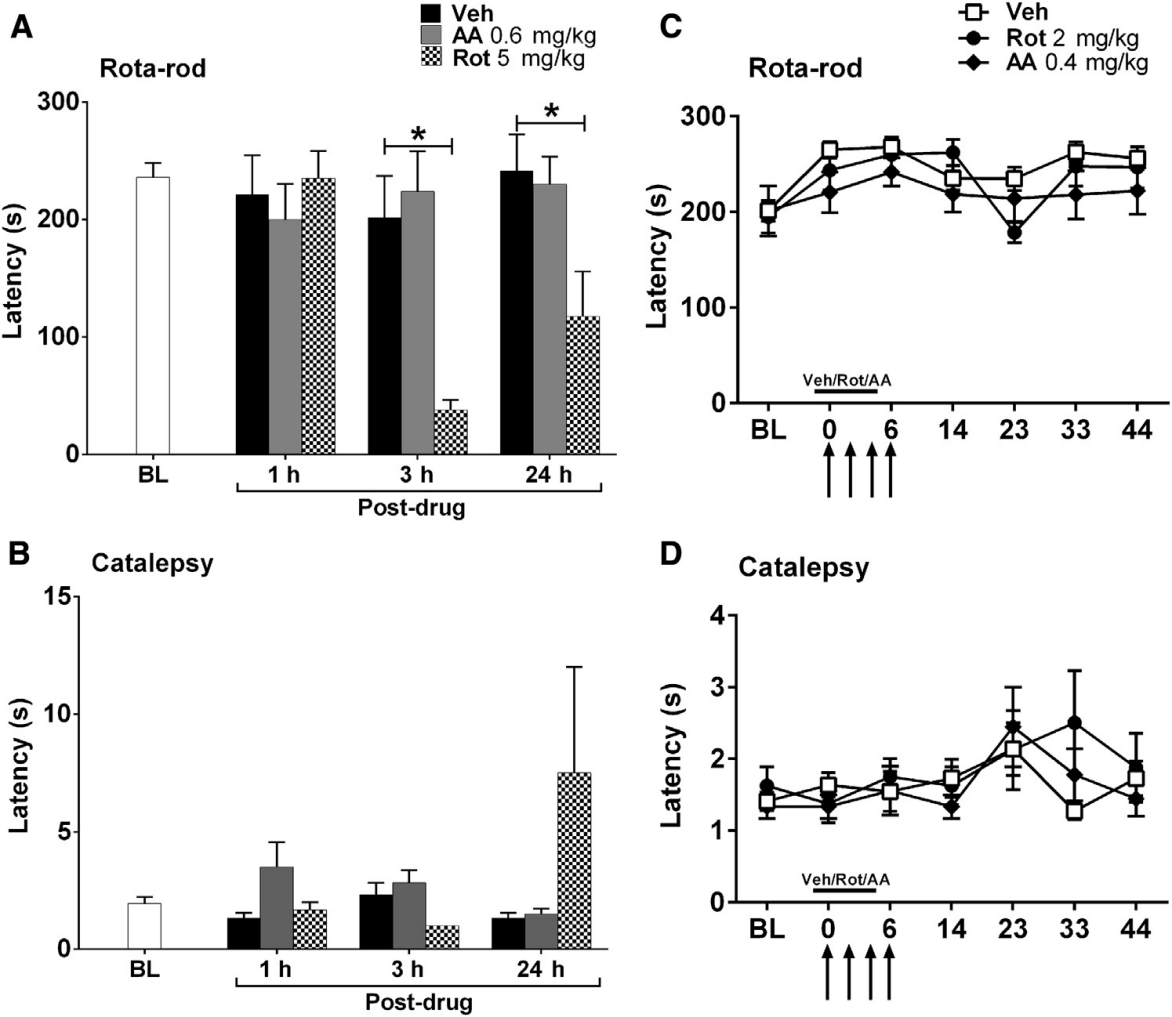
Effect of complex I or III inhibition on motor function in naive rats. All graphs show the mean ± SEM of responses. **(A, B)** The effects of high dose rotenone (5 mg/kg) and antimycin A (.6 mg/kg) on accelerating Rota-rod and catalepsy ring latencies, respectively, before (n = 18), then 1, 3, and 24 hours after administration (n = 6 per group). *P < .05, 1-way ANOVA with Dunnett post hoc comparing with the concurrent vehicle-treated group. In a separate experiment, 3 mg/kg rotenone had no effect on accelerating Rota-rod latencies compared with vehicle treatment (data not shown). **(C, D)** The effect of daily dosing of 2 mg/kg rotenone and .4 mg/kg antimycin A on days −1 to 5 on accelerating Rota-rod and catalepsy ring latencies, respectively, up to day 44 after paclitaxel initiation. Solid lines indicate periods of complex inhibitor/vehicle administration. Arrows indicate 4 injections of 2 mg/kg paclitaxel on days 0, 2, 4, and 6. n = 22 for vehicle, n = 9 for antimycin A, and n = 8 for rotenone. Veh, vehicle; AA, antimycin A; Rot, rotenone.

Since rotenone is used to induce Parkinsonian symptoms,^4^ we examined if the doses of rotenone and antimycin A used in our behavioral studies had any deleterious effects on motor function, which could therefore potentially affect withdrawal responses to von Frey stimulation. A single dose of .6 mg/kg antimycin A did not affect performance on an accelerating Rota-rod at 1, 3, or 24 hours after administration in naive rats (Fig 5A). A single dose of 5 mg/kg rotenone significantly reduced latencies at 3 hours and 24 hours after administration of rotenone but had no such effect at 1 hour compared with the concurrent vehicle-treated group (Fig 5A, *P* < .05, 1-way ANOVA with Dunnett post hoc analysis). In a similar separate experiment, a single dose of 3 mg/kg rotenone had no effect on accelerating Rota-rod performance at 1, 3, or 24 hours after administration in naive rats compared with a concurrent vehicle-treated group (data not shown). Therefore, rotenone can affect motor coordination when administered as a single dose of 5 mg/kg but with a delay of 3 hours until onset.

In addition to the Rota-rod test of motor coordination, we assessed for drug-induced catalepsy after single doses of antimycin A and rotenone. Neither .6 mg/kg antimycin nor 5 mg/kg rotenone had any significant effect on the time spent immobile on a catalepsy ring at 1, 3, or 24 hours after administration in naive rats (Fig 5B). The larger mean latency and SEM for the 5 mg/kg rotenone group at the 24-hour time point was because 1 of the 6 rats remained on the ring for 30 seconds. Thus, the effect at 24 hours after administration was somewhat skewed by the response of this one rat. In a similar separate experiment, a single dose of 3 mg/kg rotenone had no effect on the time spent immobile on a catalepsy ring at 1, 3, or 24 hours after administration in naive rats compared with a concurrent vehicle-treated group (data not shown).

We also assessed whether 7 daily injections of either rotenone or antimycin A affected motor function as used in the prophylactic dosing paradigms described earlier. Neither .4 mg/kg antimycin A nor 2 mg/kg rotenone (administered days −1 to 5 inclusive) had any significant effect on accelerating Rota-rod performance (Fig 5C) or time spent immobile on a catalepsy ring (Fig 5D) for the duration of the experiments.

## Discussion

In a series of pharmacological behavioral studies, we have assessed the effects of mitochondrial complex I or complex III inhibition on established paclitaxel-induced mechanical hypersensitivity and the development of this painful condition. Inhibition of complex I or complex III at different time points of the paclitaxel-induced pain syndrome had differential effects (discussed below) and provides further evidence of paclitaxel-induced mitochondrial dysfunction in vivo.

Complex I inhibition, with systemic administration of the maximally tolerated dose of rotenone (5 mg/kg), inhibited established paclitaxel-induced mechanical hypersensitivity at 1, 3, and 24 hours after administration. However, deleterious effects on motor coordination were also observed with this dose at 3 and 24 hours after administration. A lower dose of 3 mg/kg rotenone attenuated established paclitaxel-induced mechanical hypersensitivity at 3 hours after administration without any impairment of motor coordination. Complex III inhibition with systemic administration of the maximally tolerated dose of antimycin A (.6 mg/kg) reversed established paclitaxel-induced mechanical hypersensitivity at 1 hour after administration. This dose of antimycin A had no effect on motor coordination. Therefore, it seems that inhibition of complex I or III can reverse or attenuate established paclitaxel-induced mechanical hypersensitivity, but with the caveat that some of the effects seen after complex I inhibition may be attributed to adverse effects of motor coordination. Other studies have examined the effects of pharmacological inhibition of complex I or III on chemotherapy-induced pain behaviors assessed by paw pressure^18^ or von Frey stimulation.^47^ Peripheral administration of rotenone, by intradermal injection, significantly attenuated vincristine-induced mechanical hyperalgesia. Similar to the effects of rotenone reported here, rotenone attenuated vincristine-induced mechanical hyperalgesia for up to 3 hours in a dose-related manner.^18^ These authors also showed that peripheral administration of antimycin A significantly attenuated vincristine-induced mechanical hyperalgesia. In addition, the effects of peripheral antimycin A were dose related and lasted for 1 hour, as also observed in our study. In contrast to our findings, after systemic rotenone administration, enhancement (20–25% approximately) of paclitaxel- and oxaliplatin-induced mechanical hypersensitivity has been reported.^47^ However, these facilitatory effects on mechanical hypersensitivity were observed at 30 minutes after administration, whereas our inhibitory effects of rotenone were observed at 1 and 3 hours after administration.

In prophylactic studies, rotenone or antimycin A was administered daily for a week, either after paclitaxel administration (days 7–13) or before and during paclitaxel administration (days −1 to 5), to establish the effects of complex I or III inhibition on the development of paclitaxel-induced mechanical hypersensitivity. Rotenone had no effect on the development of paclitaxel-induced mechanical hypersensitivity in either prophylactic dosing paradigm. Antimycin A had no effect on the development of paclitaxel-induced mechanical hypersensitivity when administered after paclitaxel. In contrast, antimycin A significantly prevented the development of paclitaxel-induced mechanical hypersensitivity when administered before and during paclitaxel administration. These antinociceptive effects of antimycin A were not accompanied by any motor impairment. Therefore, only complex III inhibition, not complex I inhibition, can significantly prevent the emergence of paclitaxel-induced pain provided that complex III is inhibited before and during the exposure to paclitaxel.

A single injection of antimycin A in naive rats resulted in a significant increase in the withdrawal responses to von Frey stimulation, suggesting that complex III inhibition is normally pronociceptive. Increased withdrawal responses to von Frey stimulation have also been reported in naive mice after a single intrathecal administration of antimycin A.^24^ In comparison, we found that systemic rotenone did not alter withdrawal responses to von Frey stimulation in naive rats. These observations are also in agreement with previously published findings.^18,47^ The switch in the behavioral effects of antimycin A from pronociceptive under normal conditions to antinociceptive after paclitaxel further demonstrates that paclitaxel causes mitochondrial dysfunction in vivo because the behavioral consequences of complex III inhibition differ. Furthermore, it suggests that the precise means by which mitochondrial ROS and its modulation affects pain behavior is likely to be dependent on neuronal status. In addition, the lack of effect of antimycin A on the development of paclitaxel-induced pain when administered after paclitaxel (days 7–13) may not indicate a lack of activity per se, as one would expect a facilitatory effect under normal conditions, but it may indicate that complex III inhibition following paclitaxel is not sufficient to counteract the mitochondrial effects of paclitaxel already initiated that are causal to the development of paclitaxel-induced pain. We did observe a reversal of established paclitaxel-induced mechanical hypersensitivity 1 hour following a higher dose of antimycin A, further indicating the contribution of mitochondrial dysfunction to the maintenance of paclitaxel-induced pain.

Our main hypothesis is that paclitaxel results in an increase in ROS due to mitochondrial dysfunction, and this hypothesis is supported by evidence from studies using ROS scavengers. Previous work has shown that phenyl *N*-tert-butylnitrone, a nonspecific ROS scavenger, can attenuate the development of paclitaxel-induced mechanical hypersensitivity.^8,23^ In one study, the authors looked at whether the timing of ROS scavenger administration was important to the outcome. The results reveal that ROS scavenging on days 0 to 7, ie, at the same time as paclitaxel administration, had no effect on the development of pain, whereas if paclitaxel was given on days 7 to 15, development of pain was attenuated.^23^ This suggests that the period between the last injection of paclitaxel and the time of normal onset of pain is a crucial window in which ROS scavenging can be effective. Here, we find that complex III inhibition with antimycin A was effective when administered on days −1 to 5 but not on days 7 to 13. Collectively, this could suggest that paclitaxel causes mitochondrial dysfunction following administration but that it takes time for the effects of excessive ROS to accumulate and go beyond the capacity of endogenous antioxidant systems to result in ROS-driven pain behaviors. Such effects may underlie the coasting phenomenon seen after paclitaxel. Our data also show that modulation of mitochondria during paclitaxel exposure produces long-lasting beneficial effects; for example, the inhibitory effects of antimycin A administration on day −1 to day 5 are observed up to day 28. These data further suggest that early mitochondrial dysfunction in neurons is a key initiator of paclitaxel-induced painful neuropathy.

The role of ROS has been demonstrated in various other models of acute pain and chronic pain evoked by either inflammation or nerve injury.^16,21,22,26,31,39,40^ The effects of pharmacological modulation of the mitochondrial electron transport chain on pain behavior and ROS levels in sensory neurons have also been demonstrated.^25^ Pretreatment with intrathecal rotenone markedly inhibited *N*-methyl-D-aspartate–evoked mechanical hypersensitivity and prevented *N*-methyl-D-aspartate–evoked increases in mitochondrial superoxide levels in the superficial and deep dorsal horns of the spinal cord.^25^ A substantial body of evidence has shown that ion channels in sensory neurons can be regulated by ROS and reactive nitrogen species (for a recent review, see Gamper and Ooi^14^). By extension, as mitochondria are a major source of ROS, their dysfunction may well play a role in neuronal regulation and excitability. In isolated dorsal root ganglion neurons, oxidation of M-type potassium channels increased currents and thus suppressed neuronal excitability.^27^ In comparison, oxidation of T-type calcium channels decreased currents in dorsal root ganglion neurons, also suppressing neuronal excitability.^42^ In addition, oxidation of T-type calcium channels was associated with antinociceptive effects in vivo.^42^ In contrast, other channels such as TRPA1 have been shown to be activated by ROS in dorsal root ganglion neurons, leading to neuronal excitability and pain behaviors.^2^ Although not performed in sensory neurons, two other studies using slice preparations from different brain regions have demonstrated interesting effects of pharmacological modulation of the mitochondrial electron transport chain that could be of relevance to our data. First, in cortical pyramidal neurons, antimycin A, at a concentration shown to inhibit complex III activity by ∼20%, reduced voltage-gated calcium currents by 50%.^46^ Second, in cerebellar stellate cells, antimycin A, and to a lesser extent rotenone, increased the frequency of miniature inhibitory postsynaptic currents via recruitment of α3-containing γ-aminobutyricacid_A_ receptors.^1^ Therefore, it could be hypothesized that a decrease in voltage-gated calcium currents and/or increase in inhibitory tone at sensory synapses could underpin the antinociceptive effects of antimycin A on paclitaxel-induced mechanical hypersensitivity reported here.

In summary, we have presented the consequences of pharmacological modulation of mitochondrial ROS-producing sites, complex I and complex III, on paclitaxel-induced pain behavior. Only inhibition of complex III produced antinociceptive effects on both the development and maintenance of paclitaxel-induced pain. These studies provide further evidence of paclitaxel-evoked mitochondrial dysfunction in sensory neurons in vivo and suggest that complex III activity in particular is instrumental in this pain state.
